# From punishment to treatment: a providers’ perspective on the implementation of 2009 Rockefeller Drug Law reforms in New York

**DOI:** 10.1186/2194-7899-2-10

**Published:** 2014-05-06

**Authors:** Robert Riggs, Jim Parsons, Qing Wei, Ernest Drucker

**Affiliations:** 1 New York University, New York, USA; 2 Vera Institute of Justice, New York, USA; 3 Vera Institute of Justice, New York, USA; 4 Mailman School of Public Health, Columbia University, New York, USA; 5 John Jay College of Criminal Justice, City University of New York, New York, USA; 6 Albert Einstein College of Medicine, Montefiore Medical Center, New York, USA

**Keywords:** Rockefeller drug laws, Drug policy, Drug law reform, Treatment alternatives to incarceration

## Abstract

**Background:**

In 2009, New York reformed its “Rockefeller Drug Laws”, terminating mandatory imprisonment for many drug charges and expanding the availability of treatment alternatives to incarceration. The reforms occurred in an environment characterized by high incarceration rates, racial/ethnic disparities in drug convictions and incarceration rates, and expanded use of alternatives to incarceration. Early administrative data show a large impact on the criminal justice system. Few studies have considered the reforms from the providers’ perspective and none have sought to understand how providers are experiencing the reforms in their everyday practice.

**Methods:**

To provide a providers’ perspective, we use a qualitative, case-study approach entailing in-depth interviews with drug treatment program leaders and staff in six of the leading New York City drug programs, all with extensive experience treating court-mandated clients. Our goal was to assess treatment providers’ experiences during the reforms’ first years in effect.

**Results:**

The providers’ reports indicate that no new administrative structures or processes have been developed to foster a changed relationship between the treatment system and the criminal justice system; that the reforms failed to establish an enhanced role for treatment providers in the courts; and that client assessment, decisions on choice of treatment modalities, and program length for mandated clients continue to be dominated by criminal justice rather than clinical concerns. The providers also report some improvements in their communications and relationships with court employees involved in court-mandated cases.

**Conclusion:**

Despite some positive changes, implementation issues are potentially limiting the reforms’ ability to capitalize fully on the potential cost-savings and improvements in public health and safety that can result from the appropriate use of drug treatment as an alternative to incarceration. What appears to be occurring alongside the evolving shift from punishment to treatment under the terms of the reforms is a growing demand for treatment providers to meet the requirements of the powerful criminal justice system.

**Electronic supplementary material:**

The online version of this article (doi:10.1186/2194-7899-2-10) contains supplementary material, which is available to authorized users.

## Introduction

In an era in which state budgets across the United States have been strained by high incarceration rates, policy makers have grown increasingly receptive to the utilization of substance abuse treatment as an alternative to incarceration (ATI) for drug-using individuals charged with certain drug offenses. From the creation and expansion of drug treatment courts to changes in sentencing structures, the national trend has been arcing toward a wider availability of options in the disposition of cases involving non-violent, drug-related crimes. These changes have followed years of research showing the effectiveness of treatment and much advocacy work aimed at shifting drug policy from a punitive model to a medical or public health model.

;Perhaps no state policy change has more starkly evidenced an official move away from punishment and toward treatment than recent reforms to New York State’s “Rockefeller Drug Laws”. When Governor David Patterson signed the Drug Law Reform Act of 2009 (DLRA) into law, New York ended one of the most rigid and punitive drug policies in the nation and entered an era that, at least on paper, recognized that treating drug offenders represented a better and more cost-effective way to increase public safety. Passing the DLRA constituted an important step toward treating rather than punishing non-violent drug offenders. In NYS, the imprisonment of drug offenders has declined 37% since the 2009 legislative reforms came into effect (DCJS 2011). But the legislation’s ultimate outcomes, in such areas as the long-term effects of averting prison sentences and the success of drug treatment programs in breaking the cycle of drug use and drug offending, will largely depend upon how its changes have been implemented and how they are being carried out in practice.

Three years after the DLRA’s passage, it is clear that the impact on the criminal justice system has been dramatic; however, very little is apparent about how the reforms and their implementation have affected the substance abuse treatment system at the operational level. How are treatment providers experiencing the reforms in practice? What is their view of procedural changes related to diverting eligible defendants into treatment under the reforms? How do they see operational matters in terms of the reforms’ explicit attempt to shift from punishment to treatment? What does the new relationship between criminal justice system employees and substance abuse treatment system employees look like in the post-reform period from providers’ vantage point? Few studies have looked at drug law reform from the perspective of treatment providers, and none have specifically attempted to understand how they have experienced its implementation at the level of daily operations.

This paper provides a qualitative portrait of the DLRA’s implementation from the perspective of substance abuse treatment providers in New York City (NYC). It is based on research made possible by a Fellowship jointly administered by John Jay College of Criminal Justice and the Vera Institute of Justice^a^. The data are drawn from consultation and interviews with several experts familiar with treatment, case management, and drug law reform issues in NYC and from in-depth, semi-structured interviews with ten individuals employed by NYC-based treatment provider organizations that are established parts of the city’s ATI apparatus. It is important to emphasize that our data are not derived from a systematic random probability sample of the population of treatment providers in NYS and that the research neither aims at nor claims to provide findings that are statistically generalizable to that population. Our goal with this research was not to understand the distribution of NYS providers across pre-chosen categories. Instead, we endeavored to *uncover* the categories that are relevant to treatment providers in their experience of the implementation of drug law reforms in NYC. Understanding these categories offers insight into the reforms’ implementation from the providers’ perspective and offers information with which to generate new hypotheses and stimulate further research^b^.

## Background

New York State’s “Rockefeller Drug Laws”, passed in 1973, were the first state laws to mandate lengthy prison sentences for many drug offenders. This approach to sentencing drug offenders spread to many other states and Federal jurisdictions in subsequent years (Maggio 2006). In NYS, the proportion of the incarcerated population held on drug charges rose dramatically under these laws—from 11 percent of new commitments in 1980 to 47 percent in 1990 (DCJS 2010), and the trend throughout this period was similar nationwide.

### Rising incarceration rates

Between 1970 and 2007, the nation’s incarcerated population grew tenfold—to over two million in state and Federal facilities (Petteruti and Zeidenberg 2007)—making the United States’ incarceration rates higher than any in the world (Walmsley 2009; Warren 2008). While there are many factors contributing to this growth, research suggests that it is largely the product of rising commitments for drug offenses combined with the increasing use of mandatory minimum sentencing, as well as the role of predicate offenses in lengthening sentences for drug charges (Drucker 2002 2011; Harrison 2001; Rengifo and Wilson 2005). Of the growing number of individuals incarcerated for drug offenses nationally, 58 percent are non-violent and have no history of high-level drug charges (King and Mauer 2002).

### Racial and ethnic disparities

The increase in the use of incarceration as a response to drug offending has had a disproportionate impact on communities of color. Despite findings that rates of drug use are only slightly higher for African Americans (10.7 percent) than rates of use for whites (9.1 percent) (SAMHSA 2003), 27 percent of the increase in the African American prison population between 1990 and 2000 was attributable to drug offenses, as compared to 14 percent of the increase in the white prison population during that same time period (Beck and Harrison 2001). The racial and ethnic disparity in incarceration for drug offenses is particularly striking in NYS, where in 2001 the ratio of African American males to white males ages 21-44 incarcerated for drug offenses was greater than forty to one (Beck and Harrison 2001).

In the face of these rising levels of incarceration and the disparities involved, public support grew for reform of sentencing policies for persons convicted of drug offenses. A 2002 national opinion survey found that over 70 percent of Americans supported mandated drug treatment and community service sentences over incarceration for persons convicted of possession and low-level distribution charges (Hart Research Associates 2002).

### Alternatives to incarceration (ATIs)

ATIs for drug offenders are held out as one way to reduce imprisonment without compromising public safety, and over the last 20 years, there has been an explosion in the availability of treatment-based ATIs. One widely used ATI mechanism has been drug courts. Drug courts typically divert people facing non-violent charges from prison and jail into treatment, providing direct court supervision, coordinating treatment services, and expediting case processing. There are approximately 2,600 drug courts currently operating in the US (NIJ 2012), with 147 of these in NYS (NYS UCS 2014).

Another important method for diverting substance abusers into treatment involves sentencing options available to judges and diversion by prosecutors, for cases that are not handled by specialized drug courts. These may take the form of a sentence of substance abuse treatment as a condition of probation or as an intermediate “punishment” with no probation component attached (Warner and Kramer 2009). In a number of jurisdictions, including several in NYS, prosecutors have the option of offering treatment based alternatives as part of a plea-bargain agreement.

### Treatment and supervision

Studies have shown that community-based treatment of offenders is effective if programs are carefully designed and properly implemented (Mitchell et al. 2012; Rossman et al. 2011; Belenko 2001; Belenko et al. 2005; NIDA 2007; Pearson and Lipton 1999). Offenders who participate in community-based treatment either through drug courts, as a condition of probation or parole, or as part of a sentence imposed by a judge are subject to the demands of both the criminal justice system and the drug treatment system—two systems that do not always align in terms of their goals and methods. Indeed, there exists an inherent tension between the criminal justice model and the clinical model in managing individuals in treatment: criminal justice actors are typically more focused on monitoring compliance with treatment goals and imposing sanctions for defendants’ failures to do so; and clinicians see these “failures” as normal parts of the treatment process and assert the need for modifications of individualized treatment plans.

Marlowe (2003) points out that “public health proponents and public safety proponents may have different types of drug-involved offenders in mind” when they advocate for their preferred model, and he distinguishes between “low-risk” and “high-risk” offenders, arguing that finding the correct level of criminal justice intervention for individuals in treatment should follow from an assessment of those individuals’ risk status and their response to treatment (p. 13). He concludes, “Programs that *jointly allocate responsibility* for clients to criminal justice and drug abuse treatment professionals are in the best position to respond readily by increasing or decreasing their *coordination of efforts*, depending upon clients’ performance in the program” (p. 13, emphasis added).

In terms of joint responsibility and coordination between criminal justice and treatment, Wenzel et al. (2001) point to the potential of drug courts. They call the drug court, in its ideal form, a “bridge” between the criminal justice system and the public health system due to the collaborative links it potentially engenders between the courts, treatment providers, community-based organizations, and other social service agencies. Wenzel and colleagues studied 14 drug courts in the US and found that in nearly every case the relationships between the courts and service providers were informal and lacked systemization, with problems of information sharing and documentation evident. They note that in practice “there may be a tension between the supervisory and rehabilitation objectives of drug courts that may interfere with building bridges and access to services” (p. 250).

Research suggests that ATIs that include a high level of criminal justice supervision without a robust treatment component are ineffective in managing drug-using individuals. Petersilia and Turner (1990) studied Intensive Probation Supervision (IPS) programs in California and found that participants under intensive supervision had significantly higher rates of technical violations than those under regular supervision and that there was no significant difference in arrest rates between the two groups. They argue that “the issue of adequate drug treatment and monitoring cannot be ignored” considering that more than half of participants were in need of treatment and few received it (p. 109). Moreover, they found that significant percentages of technical violations and new arrests involved drugs. In a later study of IPSs, the authors conclude that more intensive supervision without effective substance abuse treatment had no significant impact on drug-using offenders’ subsequent criminal behavior (Petersilia and Turner 1993).

All of these factors—the relationship between commitments on drug offenses and rising rates of incarceration, the largely non-violent profile of persons arrested on drug charges, the striking racial and ethnic disparities in drug arrest and incarceration (despite minimal racial and ethnic differences in drug use), and increased public support for ATIs for this population—have contributed to recent moves toward reforming states’ drug laws as a potential method to reduce incarceration costs without threatening public safety.

### NYS 2009 drug law reform

Much as the “Rockefeller drug laws” provided a model for establishment of the national use of long mandatory sentences for drug offenses, the recent DLRA may offer an example and road map for other jurisdictions considering similar changes. After several earlier amendments to the original laws in 2002 and 2005, the NYS Legislature instituted the most significant changes to date in April 2009, when mandatory prison sentences for some drug offenses were eliminated and minimum sentence lengths were reduced for others. Table 1 provides details of the DLRA’s sentencing changes.

**Table 1:** **Summary of DLRA sentencing changes**

	***Drug conviction charge level***				
	B Felony	B Felony predicate*	C Felony predicate*	D Felony predicate*	E Felony predicate*
*Pre-DLRA Sentence options*	1-9 yrs prison; OR SHOCK^1^	3 ½ - 12 yrs prison	2-8 yrs prison; OR SHOCK^1^	1 ½ - 4 yrs prison; OR SHOCK^1^; OR Willard^2^	1 ½ - 2 yrs prison; OR SHOCK^1^; OR Willard^2^
*Post-DLRA Sentence options (changes in bold)*	1-9 yrs prison; OR jail term ≤ 1 yr; OR probation; OR judicial diversion; OR Willard^2^; OR SHOCK^1^	2 - 12 yrs prison; OR judicial diversion; OR SHOCK^1^	1½ - 8 yrs; OR jail term ≤ 1 yr; OR probation; OR judicial diversion; OR Willard^2^; OR SHOCK^1^	1½ - 4 yrs prison; OR jail term ≤ 1 yr; OR probation; OR judicial diversion; OR SHOCK^1^; OR Willard^2^	1½ - 2 yrs prison; OR jail term ≤ 1 yr; OR probation; OR judicial diversion; OR SHOCK^1^; OR Willard^2^

*With a prior *non-violent* offense.

^1^SHOCK is a 6-month boot camp program; post-reforms, SHOCK can be court ordered.

^2^Willard is a 90-day intensive treatment program; participants are under parole supervision.

In October 2009, Article 216 of the Penal Law became effective, expanding judicial discretion to offer drug court alternatives to certain addicted non-violent offenders. Article 216 gives judges the power to divert eligible defendants into treatment, even over the objection of the prosecutor. This change is particularly significant in New York City, where longstanding District Attorney led diversion programs have historically been the primary vehicles for diverting cases to treatment. In response to the passage of the DLRA, the NYS Unified Court System (UCS) engaged in “an almost year-long process to plan for and implement a case processing system that would fulfill the intent of the legislation” (NYS UCS 2009, p. 1). One key change made by the UCS during this process was the creation of Judicial Diversion Parts (JDPs) in many Judicial Districts across the state, and at least one JDP in all of the five counties of New York City (Edwards 2011).

These JDPs are responsible for judicially diverting eligible defendants away from incarceration and into treatment in compliance with the DLRA. Article 216 cases constitute an entirely new category of defendant/client, and the judicial diversion program constitutes an entirely new process for offering treatment as an ATI. The judicial diversion of Article 216-eligible defendants is thus one of the most relevant parts of the DLRA in terms of assessing its implementation from the treatment perspective.

The increased use of community-based drug treatment was explicit in the state’s DLRA planning, with $71 million being allocated through the NYS Office of Alcoholism and Substance Abuse Services (OASAS) for new treatment initiatives. However, more than half of these funds had yet to be disbursed as of September 30, 2011, and thus, some of the programs originally called for remained underfunded or unfunded, including hiring more designated court-based screeners, expanding outpatient treatment services, and increasing case-management services in the courts (CCG 2012).

### DLRA: early findings

Early findings from the NYS Division of Criminal Justice Services (DCJS 2011) show that both the number of people screened to determine their eligibility to participate in drug court and the actual number of drug court admissions of DLRA-eligible cases have increased statewide. However, much of the increase in screenings is attributable to the counties outside of NYC. While screenings in NYC increased by only 5 percent from 2008 to 2010, they almost tripled in the rest of the state. Admissions increases were also higher in the rest of the state than in NYC, with NYC admissions doubling and the rest of the state’s admissions almost tripling. This disparity is likely due to the fact that the NYC counties already had robust prosecutor-led diversion programs in place prior to the reforms. Thus, it is not necessarily surprising that NYC experienced a smaller rate increase than other areas.

While agencies are monitoring the implementation of the reforms using administrative data, mostly capturing criminal justice impacts, a very limited number of studies have sought to understand the DLRA and its implementation from the perspective of substance abuse treatment providers involved in the handling of judicially diverted cases. One survey of 28 providers was conducted in 2009 by Fluellen, Gray, and Primm just two months after the last phase of the reforms went into effect. The study gathered data on providers’ views on their relationships with members of community programs and the criminal justice system and on their perception of whether they would be able to handle the increased client load, which nearly all assumed would occur as a result of the reforms. Providers overwhelmingly indicated that their relationships with their criminal justice partners were less than ideal, with 82 percent calling the relationships either “not adequate” or “somewhat adequate;” almost all believed that the reforms inherently required a realignment of the criminal justice-treatment provider relationships (96 percent) and an expansion of relationships with other community stakeholders (93 percent). In terms of capacity to handle an influx of new criminal justice-mandated patients, 72 percent of those surveyed indicated that their agencies currently had the ability to handle this increase.

A 2012 study, carried out by the New York State Senate Standing Committee on Alcoholism & Drug Abuse and the Association for Substance Abuse Providers, surveyed 156 participants from provider organizations across NYS. Among other findings, the survey found that 47 percent of respondents had seen an increase in referrals from criminal justice entities since the reforms, while 46 percent had seen no change and 7 percent have seen a decrease. Nearly all (97 percent) of those who saw an increase in criminal justice cases reported that their organizations had not seen a concomitant increase in funding.

As states begin to follow in New York’s footsteps, crafting new sentencing rules for drug offenses, there is a need to understand the issues that have arisen for New York in the implementation of the DLRA. In this effort, the views of treatment providers are crucial. The surveys described above have increased knowledge of providers’ views on the reforms and provided valuable information. This paper expands on the currently available survey data by providing the first in-depth account of NYC providers’ *in situ* experiences of treating criminal justice-mandated clients in the wake of the DLRA’s implementation.

## Methods

As noted above, the goal of this research was not to know the distribution of the population of NYS treatment providers across a range of categories chosen in advance. Rather, we sought to discover how treatment providers are experiencing the reforms at the level of everyday practice. Our aim was thus systematically to investigate *which* categories are important to providers and impact them the most. A random probability sample and survey can help us estimate parameters when we know what to ask. Given that the DLRA represents a watershed in drug policy in the state, and potentially in the nation, and that no studies have sought to understand its implementation from the provider perspective, a valuable first step is to discover the relevant categories of interest, exactly what we attempt to do here. Qualitative research is particularly suited to this task of achieving emergent knowledge. Even a careful “single-case study can justifiably state that a particular process, phenomenon, mechanism, tendency, type, relationship, dynamic, or practice exists” (Small 2009; cf. also Glaser and Strauss p. 24; cf. also Glaser and Strauss 1967; Lofland and Lofland 1995; Lamont and White 2009).

Our methodological approach to this research is to adopt Small’s (2009) application of case study logic to in-depth interview-based studies, “such that the latter may be conceived as not small-*sample* studies but multiple-*case* studies” (p. 24; cf. also Yin 2002). Whereas the goal of survey research based on random samples is statistical representativeness, the goal of multiple-case studies is quite different: “In a case model…the collection of units is, by design, not representative; each unit has its own probability of selection; and different units are subject to different questionnaires…. If the study is conducted properly, the very last case examined will provide very little new or surprising information. The objective is saturation” (Small 2009, pgs. 24-25). This methodology enables us to generate findings that are representative not of a *population* but rather of a *phenomenon* (Luker 2008). In other words, by systematically applying case study logic and modifying the focus of our interview as we proceeded through our cases, we assert that we were able to uncover general aspects of how providers are experiencing the reforms’ implementation. How the population of NYS providers is distributed across these categories is a question for future research.

The data we present here are based on a set of cases comprising consultations and interviews with treatment experts and in-depth, semi-structured interviews with ten individuals employed by six different substance abuse treatment provider organizations in New York City. The in-depth interviews took place over the course of approximately two months, from December 6, 2011 to January 30, 2012. Each interview lasted between forty-five and seventy-five minutes. Four of the interviews were with single respondents; three were with two respondents together. All consultations and interviews were recorded with respondents’ consent and later transcribed in whole or in part.

### Case selection

We began this research by reviewing legislation, literature, reports, and other data related to New York State’s drug laws and their relationship to substance abuse treatment. The aim of this part of the formative research was to gain a broad understanding of the background and current context of drug law reform and substance abuse treatment in New York. This was an important first step in developing the categories around which our interview instrument would be based. We expanded our knowledge of the field and refined our categories by consulting with three experts in substance abuse treatment, court case management, and substance abuse treatment research. With these experts, we also discussed methods for reaching out to substance abuse treatment providers.

The recruitment strategy involved emailing key actors at substance abuse treatment providers to inquire about their willingness to participate in research designed to explain how the implementation of the 2009 drug law reforms looked from their perspective. Contact information of potential respondents was gathered both from the treatment experts and from a search of New York State’s Office of Alcoholism and Substance Abuse Services (OASAS) database of state-certified providers in New York City. The researcher sent emails to forty-one individuals working at substance abuse treatment providers in the city and followed up with phone calls to all of the organizations for which phone numbers were current.

These efforts generated responses from and conversations with four individuals. Additionally, one of the treatment experts agreed to send a recruitment email prepared by the researcher to a listserv of NYC providers who were members of a statewide provider organization. The extent of the overlap between this list and the OASAS list used by the researcher is unknown. From the bulk email sent out to the expert’s listserv, two additional responses were generated. Altogether, the responses from both of these efforts ultimately led to interviews with ten respondents.

The respondents from the six treatment organizations held various positions including therapist, program director, executive director, senior director, criminal justice specialist, and admissions director. Although there appears to be a high level of role disparity among these respondents, all of them had extensive experience in the field of substance abuse treatment, all had on-the-ground practical experience with handling court-mandated clients, and all had been working in the field before, during, and after the implementation of the DLRA. Moreover, some of this seeming disparity is an artifact of position naming conventions at various organizations. For example, titles like “program director”, “senior director”, and “criminal justice specialist” indicate positions with similar responsibilities at different agencies. The organizations at which respondents worked were certified by OASAS and were among the largest and most established components of the NYC ATI apparatus. The organizations were located in the New York City boroughs of Manhattan, Brooklyn, and Bronx. Two of them offered solely outpatient services. The other four provided both residential and outpatient services^c^.

### Building and refining the interview instrument

The interview instrument was semi-structured to 1) allow the researcher to focus on the specific categories identified during the literature and document review and refined through our first three cases of consultation with treatment experts and 2) enable respondents to expand the discussion and focus on issues important to them. The original seven categories included the following:

- The process of judicial diversion
- Screening, assessment, and treatment design in diversion cases
- Resource issues related to the reforms
- Communication with criminal justice partners
- The relationship between treatment and supervision
- Referred-client suitability
- Overall perspective on the reforms and their implementation

Although the instrument retained this general seven-part structure throughout the data-collection period of the study, we modified questions slightly as data collection proceeded and began to shift our focus onto three categories that emerged as most relevant for the providers. These categories included issues related to the *process* of receiving clients judicially diverted into treatment, the *assessment and treatment* of criminal justice mandated clients, and the *relationship* dynamics involved in collaborating with court employees in the post-reform era. It became clear that these three categories were the most relevant aspects of the reforms’ implementation from providers’ perspective. By the last few interviews, providers were virtually repeating what others had already told us about these three areas of concern, indicating that we had reached saturation on these issues.

## Results

Our research identified three key categories that represent important aspects of how treatment providers in NYC are experiencing the implementation of the DLRA. First, our interviews suggest that the reforms generated little change in the process by which criminal justice clients are processed through the courts and into treatment. This finding is especially surprising in light of the reforms explicit creation of an entirely new mechanism of judicial diversion under Article 216 of the law. Second, respondents systematically indicated that important aspects related to assessment and treatment of mandated clients continue to be dominated by criminal justice rather than clinical concerns, potentially limiting the reforms’ potential to shift NY drug policy from a punishment model to a treatment model. Third, despite these problems, many respondents reported improved relationships with their criminal justice partners since the passage of the reforms. Together, the three areas of *process, assessment and treatment,* and *relationships* encompass the main post-reform issues for the NYC treatment providers we interviewed.

### Process

Although Article 216 of the DLRA created an entirely new category of court-mandated client and a completely new court process for diverting clients into treatment, these changes occurred largely without input from and with little apparent effect on treatment providers. Significantly, many of the providers interviewed for the study were unfamiliar with the term “Article 216 cases”, a term which is routinely used by judges and other court-personnel to refer to cases that are handled using the new DLRA diversion mechanisms^d^. Providers’ unfamiliarity with this term is important because it demonstrates a continued disconnect between the courts and treatment providers and suggests a business-as-usual paradigm that is out of line with the reforms’ potential to facilitate a new spirit of collaboration between these two systems. In most instances, providers made no distinction between Article 216 diversions and other criminal justice-mandated clients, such as those coming from drug courts, parole, or probation. At the level of daily operations in provider organizations, it was as if this important part of the reforms never happened.

A program director at a large, multi-site organization that offered both residential and out-patient services, reported, “No, [I don’t know the term Article 216 case]. I know about what I see from the clients themselves who have been judicially diverted to treatment and they’re given a choice between jail/prison or treatment, whether that comes from TASC^e^ or Brooklyn Drug Court or [somewhere else]”.

A Senior Director who had extensive experience in treating criminal justice-mandated clients at an established organization reported that he did not know the term and after an explanation went on to note:

Well, we normally get referrals. Those clients either wind up on probation—they get referred to us [by probation], or they come from like DTAP^f^. So they pretty much come… from criminal justice agencies and then they come to us. I mean, we may have a walk-in, but for the most part they come from a referral source and then they come directly to us…. We get clients from drug treatment courts. We get clients from the DA’s office. So we predominately get these referrals from the judicial system.

A therapist who worked at an outpatient facility that specializes in medical therapy was similarly unfamiliar with the term: “No…I’m at sea with this…What is an Article 216?” After an explanation, he went on to note, “Well, we know what court [i.e. Manhattan Treatment Court, Brooklyn Treatment Court, etc.] because we are in contact with the case managers in the individual courts to chart the progress of the patient, but I don’t know if it’s an Article 216 or some other type of diversion”.

Among the respondents who were familiar with the term were those involved with policy work or who spent significant amounts of time either in the courts or communicating with the courts; however, even they were typically unable to distinguish between Article 216 diversions and other mandated clients at the level of daily operations. For example, a provider who was a Vice President and Director of Admissions noted the following:

You know, the diversion, the drug law reform when it first came about, it was a situation that not a lot of people had a lot of information about and it went on like that for awhile and we were getting referrals that started to trickle in at one point and even then we weren’t sure, I mean is this a diversion client? There were a lot of definitions that needed to be made and a lot of categories that needed to be made, and I think that because of that…there’s never been like a smooth transition. There’s never been like a juncture where people could say, ok, this is where the diversion clients are coming in…. It still, to this day, is sort of a murky, um, issue… We know we’re getting them. We know we’re reaching out to them, but there still isn’t…like an actual system or track in place that we can clearly say, ok these are strictly diversions.

The general inability of providers to identify Article 216 diversions partly indicates a limited use of Article 216 hearings and diversions in NYC, but it also suggests that while the DLRA generated changes in the courts aimed at identifying, screening, and processing Article 216 cases, these changes were limited to the court side of the judicial diversion process and remained largely invisible to treatment providers.

We found that the reforms have brought about little, if any, enhancement of the direct interface between the courts and treatment providers. It appears that no administrative structures or processes have been created for facilitating a new relationship between the treatment system and the criminal justice system or for establishing a larger role for treatment providers in the courts. It is true, as noted above, that new judicial diversion parts have been set up in a number of courts, that the courts have employed screeners to work in these parts, and that there is a new monitoring system in place to assess the impact of the DLRA; however, these changes all occurred *in the courts*. From the view of providers, then, an Article 216 diversion procedurally looks like any other criminal justice-mandated client, and criminal justice-mandated clients all look about the same today as they did before the reforms were passed.

Part of the reason for this may be explained by a comment made by one of the treatment experts interviewed during formative research. He noted that the reform legislation had originally included some budgetary language calling for the creation of a treatment-run program in every NY county that would be a uniform, statewide system to “direct traffic” between the courts and the substance abuse treatment system. This would have been a new infrastructure and would have provided an official role for treatment in the courts; however, the program was cut in the implementation phase, according to the expert. He noted that, as a result, “there was no one directing traffic, no change in the system. The people who were already doing things a certain way just kept doing what they were doing the same way”.

The Senior Director of Centralized Services at a multi-site provider organization explained that the failure to create a new and effective infrastructure might have been avoided if provider input had been sought in the planning phase of the implementation process: “Or even in the conceptualization of this. You know, what would be the challenges? What would be the points of intersection? What were the potential areas of breakdown as we start this whole new system of communication? That did not happen at the beginning and somehow got dropped out of the process…. Was it supposed to be part of the process?.... I don’t know, but if I had designed a process, it would’ve been”.

The fact that so many of the providers interviewed were unable to identify Article 216 cases among their client population is significant as it suggests a business-as-usual paradigm in the courts that contrasts with the goal of the reforms to engender a new spirit of communication and collaboration between the criminal justice system and the treatment system. The creation of a new category of client and a new process of judicial diversion should have made an impact on the treatment system. The fact that it appears not to have done so indicates that this important part of the reforms has been implemented in a way that excludes a role for providers.

### Assessment and treatment

Considering that the reforms sought to move New York State away from a punitive model and toward a treatment model in managing non-violent, drug-using offenders, the expectation would be that assessing and treating criminal justice defendants/clients would be governed more by clinical concerns than by criminal justice concerns in the post-reform period. It appears that some progress in this area has been made (see the Relationships section below); however, many providers interviewed expressed concern that the assessments conducted by the various screening and referring agencies in the courts are often not clinically oriented and that decisions about treatment modalities and length often seemed to be determined by criminal justice rather than clinical concerns. Many respondents noted the need for more clinicians in the initial phase of the screening and assessment process carried out in the courts.

When asked how treatment length was determined in cases involving criminal justice-mandated clients, a Director of Centralized Admissions at one large agency explained, “How long it’s gonna be comes from the criminal justice system… That’s a criminal justice decision. No, it’s not clinical at all; Not so good…because, especially if, what they’re requesting, after our assessment, we feel should be different, we’d like to have that conversation with them to discuss, you know”.

A Criminal Justice Specialist at a large and well-respected Manhattan provider discussed how, in some cases, his organization had to defer to criminal justice and referring entities on when to end the treatment of a mandated client: “We cannot release a DTAP person, even if we feel they’ve completed, until DTAP says we can let them go…until TASC says. They oversee”.

Similarly, a staff member at a multi-site organization mentioned that she had “heard of clients staying in CRs [community residences] for like a long, long time because they haven’t gotten their GED. They can’t finish their mandated requirements because this person can’t get their GED”.

Along these same lines, another treatment supervisor mentioned the lack of a clinical perspective in the initial phase of the screening and assessment process carried out in the courts and discussed the tension this created between the court’s treatment plan and his own agency’s plan:

I think mainly their assessment likely has a lot to do with offense levels…. What ends up happening is that they’re sent to us… We have a plan, but there’s also a plan at the drug court level. And the plan at the drug court level has a lot to do with sobriety. How long can this person remain sober? A lot of their plan is based on adherence to sobriety, in other words, passing urine tests. Where our plan is based on individual psychotherapy, group psychotherapy that includes a lot of education, relapse prevention, education about alcohol abuse, the neurobiological impact…. I think part of the problem is that these people [in the courts] are legal entities [rather than clinical entities].

There is an important link of this issue to the fiscal interests of the providers. Considering that the treatment organizations depend upon the court and its referring agencies for clients, which frequently constitute a significant part (and in some cases the majority) of their programs’ income, there exists a powerful incentive in the current system for providers to “go along” with decisions affecting clinical care that are made by the criminal justice entities. As a Program Director reported,

Treatment agencies in general do whatever a judge says, right? You know where your bread is buttered. So when the judge says, ‘He will go to 2 years of residential,’ and we say, ‘He’s really only appropriate for outpatient,’ we don’t argue. We sign that person up for two years of residential and they’re stuck there. It’s used as an option for incarceration.

But doing “whatever a judge says” does not necessarily imply any direct communication between providers and judges (or any official) in the current system. Instead, these communications are typically mediated by court-employed referral and case-management agents.

When asked what they would recommend to improve the type of issues discussed above, providers argued that there should be a larger role for treatment considerations in the process of deciding which cases to divert and more clinician involvement in the initial screening and assessment phases in the courts. One director of an outpatient program explained, “I would love to see more social workers in this process, in the courtroom, from the beginning of this process, because social workers have a big influence on the judge…on decisions that they make. So I would like to see more social workers in that arena when it begins”.

A Criminal Justice Specialist at a large provider agreed: “I think, if I was to be able to have a voice in it, that they should have more clinical people there making assessments instead of the criminal people in the courts. If you’re saying you want to work with the treatment part of this thing, instead of the criminal…[then there should be more clinical people in the courts]”.

Another treatment provider also felt that there was a specific need for treatment professionals to play more of a role in the early part of the assessment and screening process:

Well, I think that…some of them have them; they have what’s called a CASAC. A CASAC is a Certified Alcohol and Substance Abuse Counselor. And if on the front end, they had somebody screening them, before they get referred, then they’d be better at sorting out people’s appropriateness for different levels of treatment instead of just, ok, everybody’s going to residential. A clinician should be part of this whole process. Not everybody does that. It should be part of the process because then they’re able to do a quick assessment based upon, not just your arrest but personal history. But what happens is most judicial system [people] want to refer to residential because of the thing of custody… While it’s not a jail, but a custody thing, so they’ll refer [to residential]. In outpatient, you come and go. So it becomes more custodial a lot of times. Well, it would help with making sure this person goes to the appropriate level. I mean, I wouldn’t go to a podiatrist if I had chest pain.

A new infrastructure that included a role for treatment in key decision-making would have possibly helped to improve some of these problems with assessment and treatment. At the very least, such a new system would have put treatment providers in closer and earlier contact with actors in the criminal justice system, with the explicit goals of fostering better communication and building relationships.

Interestingly, in the absence of a systematized, uniform interface between the courts and treatment, with an *official* role for treatment in the courts, nearly all providers interviewed discussed using “Criminal Justice Liaisons”. These are provider employees responsible for spending time in the courts to advocate for defendants to be placed into their programs and for building relationships with court employees who refer and case-manage criminal justice clients. Some providers reported having these positions in place before the reforms, but others noted that they had created such positions after the passage of the reforms. For example, a senior director at an outpatient facility noted,

I think we adapted by creating the CJ [criminal justice] liaisons. You know…it was created as a result of the reforms. We saw that we would get referrals from a specific division of parole or a specific drug court but not all of them so we created this to make sure that it wasn’t out of sight out of mind. So now that I have somebody whose job it is 5 days a week to go in and out of every court in every borough we have a greater exposure and a greater presence and as a result of that, greater communication.

In other words, instead of an official, formalized system to facilitate communication and collaboration between treatment and criminal justice, individual provider agencies have created informal mechanisms to build these relationships at their own expense. However, these liaison positions still do not address providers’ concerns over the lack of clinician involvement in the assessment and screening process, since these employees are typically not social workers or CASACs and are not at all involved in assessment and screening.

### Relationships

Although many providers expressed concern over what they perceive as a disconnect between treatment and criminal justice—especially in areas such as initial assessment and decisions about treatment length and treatment modality— several reported that their relationships with their criminal justice partners seem to have improved in the years since the reforms. While not all providers expressed universal satisfaction with every aspect of these relationships, many discussed how they are now able to collaborate effectively with their criminal justice partners in treating mandated clients, a situation that was new to many if not to all providers.

When asked whether they perceived any tension between treatment and supervision in treating clients mandated by criminal justice, for example, some providers noted that this had never been an issue. One explained, “I couldn’t say that that’s been a problem. I think that if someone is noncompliant or is testing positive and we notify the court, that’s a collaborative effort to move to the next step”. Similarly, another flatly stated, “No”, without elaborating.

Other providers, by contrast, had not always experienced smooth relationships with their criminal justice partners and discussed how tension between treatment and supervision had lessened since the passage of the reforms. One explained, “Not now. In the past… But in this last year, they’re not like that anymore. They really understand. Now, you know, we go to them and we say this happened and that happened; we give a recommendation, and they kind of go along with it”.

Another provider also agreed that matters had gotten better: “My understanding is that the courts really are working towards the abstinence model. It’s not harm reduction. It’s the abstinence model and [they] have grown a little more tolerant over the years, which is helpful to us treatment people because, you know, we understand that relapse, one relapse, doesn’t necessarily mean the person needs to go back to jail or prison. And so I’m happy to see that. I think some of our clients would like to see a harm reduction model, but that’s not in the cards right now”.

Even those providers who felt a great degree of tension in their relationships with their criminal justice partners discussed how successful collaboration is sometimes achieved. A senior program officer’s comments are a case in point:

There’s a ton. I mean, the example that I heard most recently that I’m just furious about is, we’ve got a guy at [one of our facilities] who is 9 credits short of his Associate’s degree… He’s doing wonderfully. [A representative of the New York City courts] won’t let him go to school; they want him to get a job or go to voc training… The fact that they’re restricting that kind of access to education is infuriating. So yeah, there’s some tension…. [But] It depends on the facility. And it depends who the mandator is. So sometimes what happens is you immediately call the PO or whoever’s on the paper. I think it’s actually a reasonably collegial relationship… So a kid absconds, leaves because he was gambling or has a positive drug test or whatever, and then we work with the courts to get him back. They won’t remand him…. When it works right, it’s a partnering.

Often, “working right” depends upon the existence of established relationships with criminal justice representatives, as some providers explained. An employee of a large, multi-site treatment provider noted, “It sounds to me like some of the requirements and rules are really infuriating but that where there are personal relationships with the CJ folks, we’re able to work with them on a day to day basis”.

Another provider also mentioned the importance of established relationships, and his comments are worth quoting at length:

I think we’re very good at the relationship building and meeting the needs of whoever refers to us. That doesn’t mean that we’ll ever compromise treatment for a client if we feel that they need something that’s different from what they court folks are asking for…. We’ll talk on the phone. It happens all the time where a court person may say, these are the needs of this person…and we may disagree. Chances are, we mostly agree with their recommendations. We all want the same good thing for the clients. But when there is a point of tension, a point of disagreement, what we have is a conversation and we try to work pretty closely with them and more often than not we come to a very strong agreement about how we need to go forward…. That’s not to say that there isn’t tension…That’s why we have the court liaison team because sometimes the clinicians in the program don’t really know the treatment court staff as well as our court liaison staff…And what we do is we have them step in the middle. Let’s say Brooklyn Treatment Court has a question about how a client is being treated (*fictionalized example*). Now whether or not it’s good or bad, they may reach out to the program; in fact they will reach out to the program. They may or may not get the response that they’re looking for. In that case, they’ll [the court will] reach out to us, the court liaison team, and they’ll say, ‘Look is there any way you and your staff can sort of step in and address this issue for us?’ And we do that. We do that without compromising the clinical integrity of treatment but at the same time reminding the staff here that we do have a relationship. It is important that these folks get the progress reports on time and that there has to be very strong communication with the court so that they’re able to have their needs met as well.

Despite areas of disconnect between the treatment system and the criminal justice system, the relationship between the two systems, from the perspective of providers, appears to have improved since the passage of the DLRA. Providers report an increased understanding by criminal justice actors of treatment issues, such as relapse, and stress the importance of establishing professional relationships between representatives of their organizations and representatives of the criminal justice system.

## Discussion

Our specific findings concern procedural matters related to the passage and implementation of the DLRA, operational issues concerned especially with defendant/client assessment and treatment, and relationship dynamics between treatment providers and actors in the criminal justice system, including how these relationships may have changed since the passage of the DLRA. By offering treatment providers’ views of these three areas, our research provides some of the first information on how providers are experiencing the reforms in practice. These findings also enable us to begin assessing how far the reforms have gone in shifting NY drug policy from punishment to treatment.

Most notably, the absence of new administrative structures and procedures concerning contact and communication between the courts and the treatment system should be considered a hindrance to shifting the way NYC responds to drug-using offenders. Without a new system that includes a greater role for treatment in the courts from the beginning of the diversion process, NYC has been left to rely on its existing systems. This seems to have led to “business-as-usual” where the referral and communication processes are concerned. It is likely that from the courts’ perspective, this finding of business-as-usual makes little sense. As noted above, the courts did create a new judicial infrastructure in order to comply with the DLRA, namely the Judicial Diversion Parts. However, our findings indicate that drug treatment providers receiving referrals from the courts have not experienced much, if anything, in the way of change from a procedural standpoint, especially where referral and communication about the clinical aspects of the cases are concerned.

This disconnect is likely related to what one advocacy group has called the “limitations of court-directed implementation” of the DLRA (CCA 2009). In ceding control of implementing the reforms to the judiciary, policymakers have (so far) missed an opportunity to generate closer collaboration between treatment providers and the courts and to signal to the courts that treatment has a new role to play in the entire process henceforth. Collaboration between the two systems from the very beginning of implementation would have not only likely changed the way the reforms were carried out but also exemplified the type of cooperation that seems necessary in a new paradigm focused on treatment rather than punishment.

NYC has a relatively long history of providing treatment as an alternative to incarceration and has historically made greater use of ATIs than jurisdictions elsewhere in New York State. These alternatives include prosecutor-controlled programs such as DTAP, drug courts, and sentences to probation that include a treatment mandate. Throughout this history, judges, prosecutors, and case-referral and case-management agents have developed habits of practice and expectations, not to mention entrenched interests, in some cases, in the status quo. The longstanding use of diversion and the prominent role played by prosecutors in determining eligibility for drug court participation perhaps explains providers' concerns that criminal justice considerations continue to trump clinical ones in many cases. A legislative reform alone is not enough to change the practices of situated actors used to operating in established ways. Again, the importance of creating new structures and procedures in the implementation phase is relevant.

Without a new infrastructure that includes some officially recognized role for treatment in the courts, what seems to have occurred is that “people who were already doing things a certain way just kept doing what they were doing the same way”, as the expert quoted above noted. It is possible to overstate the importance of this point, for two main reasons. First, part of the tension between treatment and criminal justice is undoubtedly related to the historical separation between the criminal justice system and the treatment system, including areas where the aims of the two systems may diverge. Second, “clients” or “patients” in treatment are also “defendants” under the jurisdiction of the criminal justice system and considered criminal offenders throughout the treatment process. Thus, even a new infrastructure including an officially recognized role for treatment in the courts would still be limited in its capacity to shift the power dynamics between these two systems. However, there is growing awareness that the development of fuller collaboration aimed at bringing these two systems closer together and balancing the power relationship would be beneficial to all involved, serving both CJ and treatment interests.

The early signs of improved relationships between treatment providers and criminal justice representatives evident in our findings indicate that the reforms have already generated some positive shift of NYC’s drug policy from punishment to treatment. The comments of providers we interviewed suggest that there may be two primary avenues by which these relationships have improved. First, those comments suggesting recent general improvement in relationships and greater understanding by criminal justice actors of issues such as relapse indicate a possible “ideological rub-off” effect of the reforms. The reforms’ explicit and highly publicized intent to move from punishment to treatment may have influenced individual criminal justice actors’ behavior, perhaps warranting more lenient behavior by those already disposed in that direction and shifting the behavior of even some of those more inclined toward a punitive paradigm.

The second, and probably more significant, avenue of relationship improvement is clearly the enhanced use of treatment-employed court liaison positions since the reforms. Interestingly, rather than bringing criminal justice and treatment closer together, these positions are essentially an added layer or buffer between them. This point brings up the important issue of the divergences in practice and aims between the criminal justice system and the substance abuse treatment system. Even before the reforms, the court-employed case management and referral agencies in NYC had constituted an intermediary system between treatment and criminal justice; the treatment-employed liaisons increasingly now stand as yet another. What seems to be occurring in many cases is an intermediary communicating with an intermediary. Thus, in this case, improved relationships may have resulted not necessarily from increased understanding between the two systems but rather from the addition of another form of “lubricant” that has helped to decrease friction between them.

Our study’s findings are limited in two key ways. First, as we have discussed, our collection of cases is not a probability sample, so the providers we interviewed cannot be considered representative of NYC or NYS providers. What this means is that we do not know how these populations would be distributed across questions derived from the categories we have identified in this paper. We might observe interesting differences in particular between the NYC and NYS populations. As noted, NYC has a robust history of providing treatment as an alternative to incarceration. This means that the city already had infrastructures in place for diverting clients from the criminal justice system to the treatment system before the reforms. This is not necessarily the case in jurisdictions outside the city, especially those in the upstate area. In these areas, the newly created case processing system for diverting clients under Article 216 of the DLRA may have had a more significant impact on providers’ everyday practices. Upstate providers may not be experiencing the type of process issues identified from our case studies of NYC providers. Did upstate providers have any voice in implementing the new judicial diversion processes in their jurisdictions? Do they know and use the term “Article 216 cases” in their everyday practice? Has their communication and relationship with the courts improved in the post-reform era? What our paper has provided is valuable information with which to generate questions such as these.

Our study’s second limitation is related to the first. Our collection of cases is a non-probability “small sample” and as such does not allow generalization. The size of a sample needed for statistical generalizability depends, among other factors, on how pre-chosen variables are distributed, whether a study aims to estimate a proportion or find a causal relationship, and the total number of pre-selected variables in the model. Hence, if statistical generalizability were the key criterion of validity, then the type of in-depth, open-ended, evolving qualitative interviews we conducted in our study of cases could never be carried out no matter how many people were interviewed. A future study that derives questions from the categories we have identified would need to be conducted on a much larger, random sample of providers in order to determine generalizability to the population.

Our research contributes to knowledge of the drug law reforms by presenting (for the first time) the voices of providers and their views of the evolving dynamics of treatment in the wake of the passage of the DLRA. These providers have had extensive experience in treating criminal justice-mandated clients at established treatment organizations in NYC and did this work throughout the period before the passage and implementation of the DLRA. And despite its limitations, our research identifies key issues of inquiry, delineates areas for further investigation, and offers specific data with which to generate hypotheses and stimulate further research.

## Conclusion

In coming to conclusions regarding our research, it is worthwhile to look back to 2009 when the reforms were passed in order to reiterate that their explicit intent was to shift New York’s drug policy from one focused on punishment to one committed to treatment. As Senator Ruth Hassell-Thompson noted at a press conference following the signing of the DLRA into law, “We are now shifting resources to treat drug addiction as a medical problem…. Study after study shows that our policies will make our communities safer and save taxpayers millions of dollars” (NYSGO 2009). Others echoed this same sentiment, as Governor Patterson’s comments on the day of the DLRA’s passage indicate: “Today, we have succeeded. With the stroke of a pen, the Rockefeller Drug Laws will end…. But to be successful we must not only overhaul the drug laws, we must also provide an infrastructure to ensure that we successfully rehabilitate those who are addicted with programs…[that exemplify] our approach to focus on treatment, not punishment” (NYSGO 2009). The overarching issue our study raises concerns the extent to which the reforms have effected the type of changes many originally hoped they would.

Clearly, treatment as a response to drug enforcement is increasingly supplanting punishment in NY under the terms of the DLRA. Statewide administrative data show that incarceration of drug offenders is down and screenings and admissions to drug courts are up since the reforms’ passage. For most providers, especially those that are publicly funded, contact with the criminal justice system is ubiquitous and now a dominant reality of substance abuse treatment. While some elements of the relationship between these two systems have been in place for years, the volume of mandated clients and the significance of the programs’ reliance on the criminal justice system for business represent a fundamental shift in the dominant conditions of treatment and alter the ethical framework for treatment itself. In other words, shifting drug policy from punishment to treatment under the DLRA has meant not only increased use of treatment as an alternative to incarceration but also changes in the practice of treatment itself.

It is now possible to see early signs of the emergence of a new model in which treatment professionals and programs accommodate to and collaborate closely with the criminal justice system. On one hand, this new model represents an increased willingness by criminal justice actors to cede to the recommendations of treatment professionals. On the other, it indicates an increasing necessity for treatment professionals to subordinate the traditional demands of treatment to the requirements of the powerful criminal justice system.

The passage of the DLRA has generated positive changes in terms of shifting New York’s response to drug offending, but implementation issues have narrowed the degree of this shift. It is too early to tell whether the limited role of treatment providers in determining which defendants should be referred to treatment as an ATI, the types of treatment that they receive, and/or appropriate levels of criminal justice supervision will negatively impact the number of people who successfully complete treatment compared to the number who ultimately enter custody following failure to meet the terms of their treatment supervision. It is already clear that, under the terms of the DLRA, we are seeing an evolving process in which a shift from punishment to treatment is occurring alongside a growing demand for treatment providers to meet the requirements of the criminal justice system.

## Endnotes

^a^The views expressed in this paper are solely the authors’ and do not reflect the views of either John Jay College or the Vera Institute of Justice.

^b^We discuss these issues in more depth in our methods section.

^c^The information provided about the sample and the respondents’ organizations is deliberately vague in order to protect their anonymity. It should also be noted that providers spoke as individuals, not as representatives of their organizations.

^d^In 2009, Article 216 of the Penal Law went into effect as part of the DLRA, giving judges, for the first time, the power to divert clients into treatment without the consent of the prosecutor. The Unified Court System created an entirely new case processing system and new “Judicial Diversion Parts” in the courts to fulfill this part of the legislative reforms. An Article 216 case refers to a defendant/client diverted into treatment through this newly created mechanism. (See the *NYS* 2009 *Drug Law Reform* section on page 8 for more information).

^e^TASC, or Treatment Alternatives for Safer Communities, is an outsourced referral and case-management agency responsible for monitoring defendants/clients diverted into treatment.

^f^DTAP, or Drug Treatment Alternative to Prison, is a prosecutor-run program located in 3 boroughs of NYC. The programs began in Brooklyn in 1990 and expanded to Manhattan and Queens in 1992.

## Authors’ original submitted files for images

Below are the links to the authors’ original submitted files for images. Authors’ original file for figure 1

## Abbreviations

ATI: Alternative to incarceration
CASAC: Certified alcohol and substance abuse counselor
CJ: Criminal justice
DLRA: Drug law reform act of 2009
DTAP: Drug treatment alternatives to prison, a prosecutor-led diversion program operating in three NYC boroughs
JDP: Judicial diversion parts, newly created court parts created under the DLRA
NYC: New York City
NYS: New York State
OASAS: New York State office of alcoholism and substance abuse services
TASC: Treatment alternative for safer communities, an outsourced case-management agency employed by the NYC courts
UCS: New York State unified court system.
