# Supplementary material for: From punishment to treatment: a providers’ perspective on the implementation of 2009 Rockefeller Drug Law reforms in New York
Source: Health Justice. 2014 May 6;2:10. doi: 10.1186/2194-7899-2-10 (PMC5151789; doi:10.1186/2194-7899-2-10)
Supplement: Supplementary file 1 — Authors’ original file for figure 1 [file 40352_2013_15_MOESM1_ESM.pdf]

Table 1: Summary of DLRA Sentencing Changes

	Drug Conviction Charge Level				
	B Felony	B Felony Predicate*	C Felony Predicate*	D Felony Predicate*	E Felony Predicate*
Pre-DLRA Sentence Options	1-9 yrs prison; OR SHOCK[1]	3 ½ - 12 yrs prison	2-8 yrs prison; OR SHOCK[1]	1 ½ - 4 yrs prison; OR SHOCK[1]; OR Willard[2]	1 ½ - 2 yrs prison; OR SHOCK[1]; OR Willard[2]
Post-DLRA Sentence Options (changes in bold)	1-9 yrs prison; **OR jail term ≤ 1 yr; OR probation; OR judicial diversion; OR Willard[2];** OR SHOCK[1]	**2** - 12 yrs prison; **OR judicial diversion; OR SHOCK[1]**	**1½** - 8 yrs; OR **jail term ≤ 1 yr; OR probation; OR judicial diversion; OR Willard[2];** OR SHOCK[1]	1½ - 4 yrs prison; **OR jail term ≤ 1 yr; OR probation; OR judicial diversion**; OR SHOCK[1]; OR Willard[2]	1½ - 2 yrs prison; **OR jail term ≤ 1 yr; OR probation; OR judicial diversion**; OR SHOCK[1]; OR Willard[2]

* With a prior *non-violent* offense.
[1] SHOCK is a 6-month boot camp program; post-reforms, SHOCK can be court ordered.
[2] Willard is a 90-day intensive treatment program; participants are under parole supervision.
